# Dynamic modeling of transcriptional gene regulatory network uncovers distinct pathways during the onset of Arabidopsis leaf senescence

**DOI:** 10.1038/s41540-018-0071-2

**Published:** 2018-08-31

**Authors:** Bharat Mishra, Yali Sun, TC Howton, Nilesh Kumar, M. Shahid Mukhtar

**Affiliations:** 1 Department of Biology, University of Alabama at Birmingham, 1300 University Blvd., Birmingham, AL 35294 USA; 2 Nutrition Obesity Research Center, University of Alabama at Birmingham, 1675 University Blvd., Birmingham, AL 35294 USA

## Abstract

Age-dependent senescence is a multifaceted and highly coordinated developmental phase in the life of plants that is manifested with genetic, biochemical and phenotypic continuum. Thus, elucidating the dynamic network modeling and simulation of molecular events, in particular gene regulatory network during the onset of senescence is essential. Here, we constructed a computational pipeline that integrates senescence-related co-expression networks with transcription factor (TF)-promoter relationships and microRNA (miR)-target interactions. Network structural and functional analyses revealed important nodes within each module of these co-expression networks. Subsequently, we inferred significant dynamic transcriptional regulatory models in leaf senescence using time-course gene expression datasets. Dynamic simulations and predictive network perturbation analyses followed by experimental dataset illustrated the kinetic relationships among TFs and their downstream targets. In conclusion, our network science framework discovers cohorts of TFs and their paths with previously unrecognized roles in leaf senescence and provides a comprehensive landscape of dynamic transcriptional circuitry.

To elucidate the dynamic network modeling and simulation of the onset of senescence, we constructed a computational pipeline (Fig. 1a). It integrates co-expression networks, network topology analyses, TF-promoter associations and microRNA (miR)-target interactions. Subsequently, our platform models transcriptional regulatory dynamics using Scalable Models for the Analysis of Regulation from Time Series (SMARTS).^1^ Finally, it simulates the kinetic relationships among TFs and their downstream targets by employing Standardized Qualitative Dynamical systems (SQUAD)^2^ that combines Boolean and ordinary differential equation (ODE) models (Fig. 1a). Utilizing this platform, we first analyzed a high-resolution time-series senescence dataset spanning from four-day-old seedlings to 30-day-old leaves that are undergoing senescence.^3^ We compared the total number of differentially expressed genes (DEGs) and emergent genes, *i.e*. first significant change in the expression gradient, at each time point. While we found approximately 475 DEGs during each sampling time point, we observed a progressive increase in the number of emergent genes over time with maximum number of 1,317 emergent genes at day-30 (Supplementary Fig. S1a; Supplementary Table S1). This analysis allows us to identify phase-specific DEGs and a set of genes that play a role at the onset of leaf senescence.

**Fig. 1:**
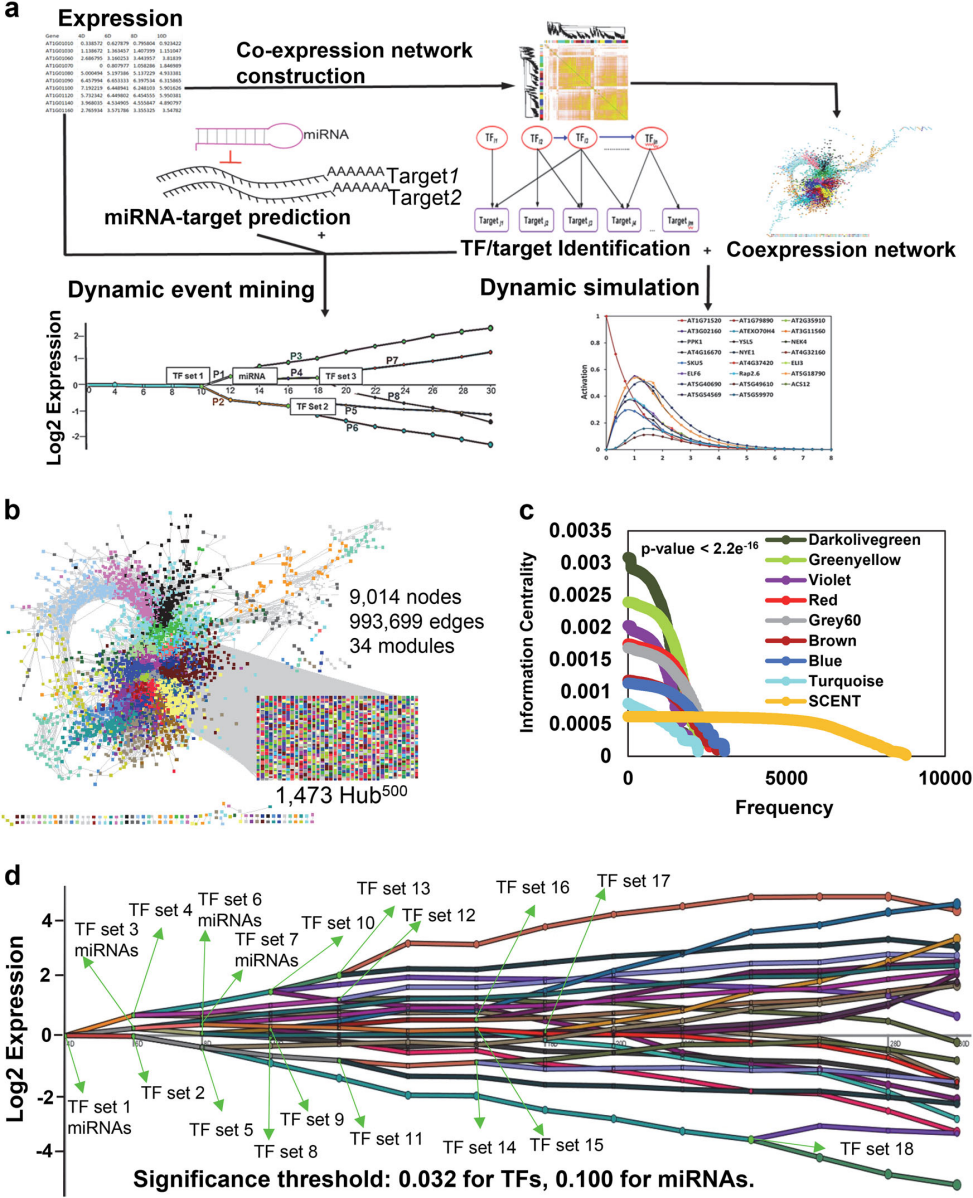
Dynamic modeling of transcriptional gene regulatory network and regulated pathways during Arabidopsis leaf senescence. **a** Computational pipeline for dynamic modeling of transcriptional gene regulatory network (GRN) and regulated pathways during the onset of Arabidopsis leaf senescence. Major components of this platform including expression data acquisition, co-expression analyses, transcription factor (TF)-targets and microRNA (miR)-target identification, dynamic transcriptional modeling, and network simulations are demonstrated in sequential steps. **b** A weighted co-expression network with weight ≥ 0.75, which was constructed by weighted co-expression network analysis (WGCNA) containing 9,014 co-expressed (nodes) and 993,699 interactions (edges), representing the Senescence CoExpression NeTwork (SCENT) was visualized in cytoscape. Nodes in different colors represent diverse modules in SCENT. 1,473 nodes with ≥ 500 connections (Hub^500^) are spread throughout different modules. **c** The information centrality (IC) distributions, path length structural centrality measure, of seven modules and SCENT revealed that darkolivegreen, greenyellow and violet modules have highest IC values. The Student's t-test (p-value < 2.2e^−16^) was applied to test the significance of ICs of all modules against SCENT. **d** Reconstructed responsive dynamic regulatory events for senescence network were modeled by SMARTS (Scalable Models for the Analysis of Regulation from Time Series). Modeled TF-targets and miR-target gene interaction, senescence gene expression and miRs expression values were utilized for event mining. The colored lines represent clustered genes based on their expression patterns. The green nodes denote splits between sets of genes that are regulated mutually until a particular interval. Forty-two different paths modeled by 18 different groups of TFs and several miRs are demonstrated as bifurcations. The dynamic activated pathways regulated by TFs and miRs were generated by TAIR Gene Ontology function, represented by a particular color with path numbers. The significance threshold was set to 0.032 for TFs and 0.10 for miRNAs regulation

To determine senescence-associated common gene signatures, we performed a weighted gene co-expression network analysis (WGCNA)^4^ in leaf senescence using two independent datasets encompassing 11,453 nuclear-encoded DEGs and 2,226 antisense DEGs. The resulting two co-expression networks are designated as Senescence CoExpression NeTwork (SCENT; 9,014 nodes and 993,699 edges; Fig. 1b; Supplementary Table S2) and Antisense Senescence CoExpression NeTwork (ASCENT; 1,158 nodes and 16,137 edges; Supplementary Fig. S5b), respectively. While the details on network analyses pertinent to ASCENT are described in Supplementary Information (Supplementary Fig. S5-S8), SCENT exhibits properties of a scale-free network (*R*^2^ > 0.6; a power-law degree distribution). Moreover, SCENT displays high mean connectivity as shown by topological overlap mapping metric (TOM) plot (Supplementary Fig. S1b). Based on cluster coefficient and connectivity of genes, we extracted 33 modules encompassing a minimum of 100 highly correlated genes that are differentially expressed during leaf senescence in SCENT (Supplementary Fig. S2). Turquoise and darkolivegreen are the largest and the smallest modules containing 1001 and 114 genes, respectively (Supplementary Table S2). A total of 366 genes were not assigned to any of the 33 modules, thus classified to a “gray” (or insignificant) 34th module (Supplementary Table S2). We observed that seven modules (darkolivegreen, greenyellow, violet, red, grey60, brown, and blue) exhibit heightened connectivity and average cluster coefficient compared to the other modules or SCENT as a whole (Supplementary Fig. S2b and c, Supplementary Table S2).

In network science, diverse centrality measures lead to the discovery of most influential nodes within a network.^5–8^ We analyzed degree (number of connections of a node), shortest paths (shortest distance between two nodes) and information centrality (propagation of information between vertices) for the nodes in SCENT and above mentioned seven modules. We discovered that information centrality and average shortest paths are significantly higher in seven modules compared to other modules or SCENT as a whole network (Fig. 1c; Supplementary Fig. S3a and b, Supplementary Table S2). Additionally, five modules (greenyellow, darkolivegreen, red, grey60, and blue) possess the majority of hub^500^ nodes in SCENT (Fig. 1b, Supplementary Table S2). Functional analyses in these seven modules revealed that most of genes in SCENT are involved in protein phosphorylation, response to salt stress, response to cold, oxidation-reduction process, protein folding, response to water deprivation and abscisic acid-activated signaling pathway followed by protein ubiquitination (Supplementary Fig. S4, Supplementary Table S3). Intriguingly, we discovered that hub^500^ nodes are significantly enriched in TFs, suggesting a central role of these key proteins in the co-regulation of their partners in SCENT (Hypergeometric test; *p* value = 7.198e^−5^).

Subsequently, we modeled our co-expression network, SCENT, to elucidate the intricate transcriptional relationships between the TFs and their target genes (TF-target association network; Fig. 2a, Supplementary Table S4). Intriguingly, we revealed the functions of 220 TFs in leaf senescence using our computational pipeline compared to previously reported 94 TFs in Woo et al.^3^ (Supplementary Fig. S9, Supplementary Table S4). Within SCENT, SMARTS highlighted major bifurcation events where the expression of a subset of genes diverged from the rest of the genes, and identified TFs potentially responsible for them. This analysis modeled 42 different paths that are governed by 18 different groups of TFs and several miRs (Fig. 1d, Supplementary Table S5). Taken together, this analysis inferred previously undiscovered dynamic and phase-specific transcriptional regulatory models that vastly expanded our horizons on the structural architecture and circuitry mechanisms in leaf senescence.

**Fig. 2:**
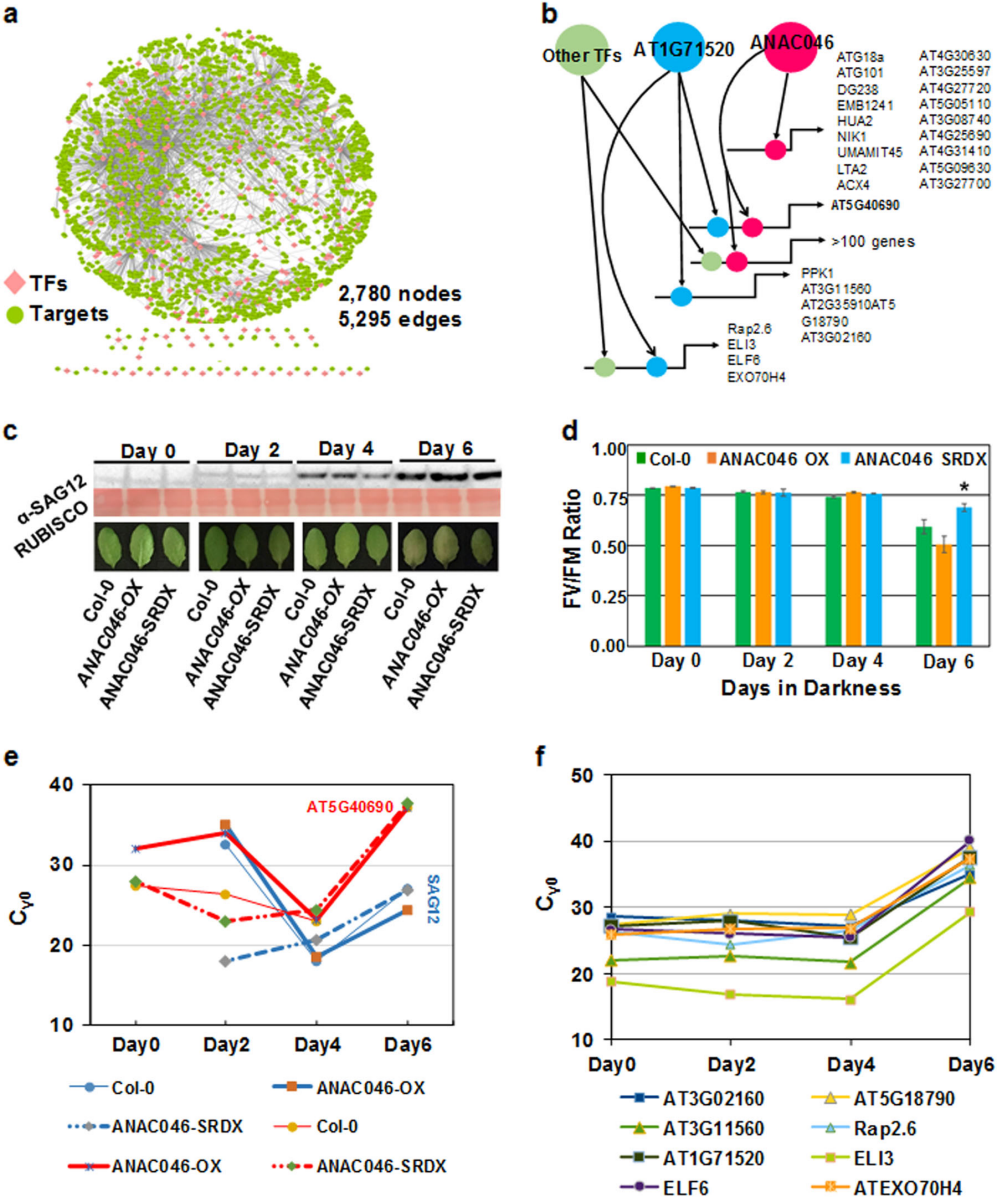
**a** Senescence CoExpression NeTwork (SCENT) was integrated to transcription factors (TFs)-targets relationships to determine the gene regulatory network. The gene regulatory network is composed of 2,780 nodes and 5,295 edges among 220 TFs and 2,560 target genes. **b** Schematic representation of target genes regulated by only ANAC046 or AT1G71520, as well as combination of ANAC046, AT1G71520 and other TFs simulated by SQUAD (Standardized Qualitative Dynamical systems). The simulated steady state regulatory network trajectory plots were modeled according to logical connectivity including partial activation of nodes. The change in activation state of TFs (ANAC046 and AT1G71520) from 0 to 1 simulates the resulting trajectory plots of their putative target genes according to the connectivity of partial activated nodes. **c** Eight representative leaves from four different plants per time point of the indicated genotypes were subjected to dark-induced senescence. Western blot was performed using α-SAG12 antibody at the indicated time points. Ponceau S stain was used to detect RUBISCO (loading control). **d** Fv/Fm measurements were taken on detached leaves (*n* = 8) from the corresponding day of dark-induced senescence for the indicated genotypes. The Student's t-test was applied to check the significance between the mutant lines and Col-0. (* indicates a *p*-value of 0.03). **e** C_γ0_ values represent the kinetic activities of SAG12 and AT5G40690 in leaf tissues undergoing dark-induced senescence at the indicated time points in Col-0, ANAC046-OX (overexpressor) and ANAC046-SRDX (knockout). **f** Varied C_γ0_ values reflect different kinetic activities of AT1G71520 and its downstream genes *ELI3*, *AT3G11560*, *AT3G02160*, *Rap2.6*, *ATEXO70H4*, *AT5G18790*, and *ELF6* at the indicated time points in Col-0

To simulate the impact of transcriptional activities on their target genes, we performed SQUAD simulations on an integrated network that contains co-expression network and TF-target association data (Fig. 2a). For downstream detailed simulations and experimental validations, we selected two Hub^500^ TFs (ANAC046, and previously uncharacterized DREB; A-5 of ERF/AP2 factor; AT1G71520) that regulate gene expression at different phases of senescence. ANAC046 co-regulates with 137 genes of which 102 nodes exhibit hub^500^ property in SCENT (Fig. 2b), whereas AT1G71520 co-regulates with 22 genes (Fig. 2b). Among them, 10 genes have been previously reported to be involved in autophagy or senescence-associated processes.^9^ The activation of ANAC046 and AT1G71520 over time was used as an index to measure the behavior of its putative downstream target genes (Supplementary Fig. S10b and c; Supplementary Table S6). This analysis displayed varied levels of activation for these downstream target genes. To validate these simulations, we subjected wild-type Col-0, ANAC046-SRDX (*anac046* mutant) and ANAC046-OX (ANAC046 overexpressor)^10^ plants to dark-induced senescence. We observed differential accumulation of SAG12 transcript and protein over time in different genotypes (Fig. 2c, e, Supplementary Fig. S11a). These data corroborate with F_v_/F_m_ quantification (Fig. 2d). Finally, qRT-PCR and mathematical modeling verified the activation of selected genes via ANAC046 or AT1G71520 by Cγ0 method (Fig. 2e, f, Supplementary Fig. S11b-d). In summary, our network science framework identified most influential nodes within the senescence network (Supplementary Discussion). Network modeling and simulations predicted functional cohorts of co-regulated genes, revealed the complexity of transcriptional regulatory machinery in leaf senescence and discovered innovative pathways during the onset of senescence.

## Electronic supplementary material

Supplemental Information

Table S1

Table S2

Table S3

Table S4

Table S5

Table S6

Table S7

## Data Availability

All data generated or analyzed supporting the results reported in this published article (and its Supplementary Information files).

## Electronic supplementary material

**Supplementary information** accompanies the paper on the *npj Systems Biology and Applications* website (10.1038/s41540-018-0071-2).
