# Supplementary material for: Dynamic modeling of transcriptional gene regulatory network uncovers distinct pathways during the onset of Arabidopsis leaf senescence
Source: NPJ Syst Biol Appl. 2018 Aug 31;4:35. doi: 10.1038/s41540-018-0071-2 (PMC6119185; doi:10.1038/s41540-018-0071-2)
Supplement: Supplementary file 1 — Supplemental Information [file 41540_2018_71_MOESM1_ESM.pdf]

Title

Dynamic modeling of transcriptional gene regulatory network uncovers distinct pathways during the onset of Arabidopsis leaf senescence

Bharat Mishra[1] Yali Sun[1], TC Howton[1], Nilesh Kumar[1] & M. Shahid Mukhtar[1,2*]

Affiliations
[1] Department of Biology, University of Alabama at Birmingham, 1300 University Blvd., Birmingham, AL 35294, USA
[2] Nutrition Obesity Research Center, University of Alabama at Birmingham, 1300 University Blvd., Birmingham, AL 35294, USA

***Correspondence**
M. Shahid Mukhtar
University of Alabama at Birmingham
Campbell Hall 464,
1300 University Blvd.
Birmingham, AL 35294-1170USA
smukhtar@uab.edu
Tel: +1-205-934-8335

Supplementary Information

Supplementary Methods and Notes

Gene expression analyses reveals important emergent DEGs

The primary data source of this study is a reproducible high-resolution Hiseq2000 time course transcriptomic expression data generated by Woo et al.[1], retrieved from NCBI's Gene Expression Omnibus[2] with the accession number GSE43616. The FPKM dataset was pre-processed, log_2 transformed and fold changed with respect to day 4. We performed differentially expressed genes (DEGs) analysis as described by Woo et al. 2016. These log_2 transformed expression profiles generated with expression parameters; >1 for up-regulated genes and <-1 for down-regulated genes. This produces a total of 11,453 differentially expressed genes (DEGs) for senescence. Cumulative and emerging DEGs are identified at each time point. We detected that cumulatively 475 genes are differentially expressed irrespective of time course from day 6 to day 30 (Supplementary Fig. 1a; Supplementary Table S1). While, several DEGs are emerging at each time point ranging from 475 DEGs to 1,317 DEGs at day 6 and day 30, respectively. We observed that emergent DEGs count follow a steep increase from day 6 to day 14, and day 16 to day 30 in two distinct patterns. However, there is a decrease in number of emergent DEGs at day 16 (291 DEGs) compared to day 14 (1,217 DEGs) and day 18 (618 DEGs) (Supplementary Fig. 1a; Supplementary Table S1). For antisense transcripts, we found approximately 600 DEGs during each sampling time point irrespective of time course from day 6 to day 30, We observed that emergent genes denoting the number of DEGs decreasing from day 6 until day 18 and begin to increase with maximum number of emergent genes at day 30 (Supplementary Fig. 5a; Supplementary Table S1). This uncertain decrease in emergent DEGs at day 16 might be due to transition of developmental stage from growth to maturation (G to M) and maturation to senescence (M to S). Importantly, we discovered that at least 4.17% of the emerging DEGs are present in the cumulative list of DEGs at any time point (Supplementary Fig. S1a, Supplementary Fig. 5a, and Supplementary Table S1).

Weighted gene co-expression network construction identified significant modules within two different senescence networks

The availability of high-resolution, large-scale transcriptome datasets enables co-expression network analysis to identify clusters of highly correlated genes which are potentially co-regulated in response to a physiological condition within a cell[3]. Thus, co-expression networks have huge potential for identification a set of genes, which might participate in a common biological process. To determine senescence associated common gene signatures, we implemented a correlation-based R package; weighted gene co-expression network analysis (WGCNA)[4,5] on two subsets of dataset with 11,453 DEGs representing overall senescence process and 2,226 DEGs representing expression of antisense transcripts. Specified complexity of the multi-course dataset, exhausting a hard threshold would result in the loss of information and may affect the sensitivity too[5]. Hence, a soft-threshold power of 18 and 12 along with a scale-free

model fit index R^2 > 0.6 and 0.83 was utilized for maximum scale-free topology, preserving a high mean connectivity and rejecting lesser correlations for nuclear genes and antisense transcripts, respectively. The connectivity network topology is indicated by their location in a dendrograms (Supplementary Fig. 1a and 6a). To ease the complete network analysis, genes are clustered into co-expressed "modules". The module in WGCNA are assigned by a flexible process, permits to affect the least number of features confined in each module by semi-programmed cutting of the dendrogram and is denoted by a unique color. We created an elementary WGCNA network utilizing flashClust() and cutreeDynamicTree() algorithms, incorporating all cleaned expression values. The resulting ASCENT (Antisense Senescence CoExpression NeTwork) and SCENT (Senescence CoExpression NeTwork) networks were generated (Fig. 1B and Supplementary Fig. 5b).

In case of ASCENT, an undirected weighted network composed of five modules of correlated genes was achieved with minimum module size >50 genes for senescence antisense transcripts (Supplementary Fig. S5b, Supplementary Table S2). The turquoise being the largest module containing 848 genes followed by blue, brown, yellow and green with 488, 72, 71 and 53 genes respectively. Based on cluster coefficient and connectivity of genes, a total of 694 genes were not assigned to any of the five modules thus supplemented to a ‘grey’ (or insignificant) module (Supplementary Fig. S5b and S6a). Similarly, the modules were also generated for SCENT. The minimum module size ≥100 for 11,453 overall senescence dataset in SCENT. The WGCNA identified 34 different modules; turquoise being the largest and darkolivegreen being the smallest module with 1001 and 114 genes respectively (Fig. 1b). The topological overlap mapping metric (TOM) heatmap verifies the clustering of like-regulated genes in each module based on hierarchical clustering for both ASCENT and SCENT (Supplementary Fig. S1b and 5c). The intensity of yellow coloring indicates highest strength of correlation between pairs of gene followed by pink. Finally, for network topological analyses and visualization the weighted undirected co-expression network was extracted utilizing a hard threshold of weight ≥0.75 and exported into Cytoscape v. 3.4[6] by exportNetworkToCytoscape function.

Co-expression network module analyses

The resulting ASCENT and SCENT were utilized for further network analyses. ASCENT represents 1,158 co-expressed genes (nodes) with 16,137 interactions (edges) (Supplementary Fig. S5b, Supplementary Table S2), while SCENT represents 9,014 co-expressed genes with 993,699 interactions (Fig. 1b, Supplementary Table S2). ASCENT and SCENT were further analyzed for network topology measures such as degree, information centrality (IC) and shortest path by cytoscape[6] and Python-based NetworkX[7], respectively. Based on cluster coefficient and connectivity of each module (Supplementary Fig. S6b and S6c, Supplementary Table S2), we focused the analyses on turquoise and blue modules for ASCENT (Supplementary Fig. S6c), and seven different modules in SCENT (darkolivegreen, greenyellow, violet, red, grey60, brown, and blue; Supplementary Fig. 2b and c). The degree of a node is the number

connections to the particular network (Supplementary Table S2), while information centrality is a substitute of closeness centrality centered on definite resistance amid nodes in a specified network to determine the course of information starting one to other node built on length of largest subnetwork of a network[8]. It is found that Blue module genes have significantly higher degree than turquoise module genes (Supplementary Fig. S5b).

Additionally, we computed information centrality with weighted parameter (*i.e.* all edges are treated as per their actual weights). The information centrality *IC*(*i*) for node *i* in a graph G is calculated as:

$$IC(i) = \left[\frac{1}{n}\sum_{j}\frac{1}{I_{ij}}\right]^{-1}$$

Here, n is the total count of nodes and $I_{ij} = \left(r_{ii} + r_{jj} - r_{ij}\right) - 1$, r_{ij} is a component of R matrix. D is a weighted degree diagonal matrix for each node and J is a matrix consisting of 1 for all elements. Therefore, $R = \left(r_{ij}\right) = [D - A + J] - 1$. Mathematically, $I_{ii}$is well-defined as infinite. Hence, $\frac{1}{I_{ij}} = 0$.

The information centrality distribution of ASCENT reveals that the blue module has significantly higher information centrality compared to turquoise and ASCENT (Supplementary Fig. S7a). Furthermore, we also calculated average information centrality of the largest component of the network. It is observed that blue module has highest average IC (0.0069) followed by 0.0027 for turquoise module, while average IC for ASCENT was only 0.0009 (Supplementary Fig. S7b). Thus, our data advocates that antisense Arabidopsis leaf senescence transcripts relies on collective genes of blue and turquoise modules to spread the information through DEGs. Also, the shortest paths between blue module and turquoise module genes were calculated to determine total numbers of shortest paths navigate through a node. Shortest path distribution reveals that genes in the blue module are closer to each other than turquoise and ASCENT (F.test, P= 0.020970693 between Blue and ASCENT, F.test, P= 0.295933097 between turquoise and ASCENT) (Supplementary Fig. S7c). Thus, any stimuli can travel faster in blue module than turquoise module.

Similarly, the information centrality distribution of SCENT reveals that the darkolivegreen, greenyellow, violet, red, grey60, brown, blue modules have significantly higher information centrality compared to turquoise and SCENT (Fig. 1c). Furthermore, we also calculated average information centrality of the largest component of the network. It is observed that darkolivegreen module has significant highest average IC (0.0022) followed by 0.0018 for greenyellow and 0.0014 for violet modules while average IC for SCENT was only 0.00049 (Supplementary Fig. S3a). Thus, our data suggests that Arabidopsis leaf senescence relies on collective genes of darkolivegreen, greenyellow, violet, red, grey60, brown and blue modules to spread the information throughout nuclear DEGs. Also, the shortest paths between all

seven modules genes were calculated to fine the total count of shortest paths navigate through a node. Shortest path distribution reveals that genes in the darkolivegreen, violet, greenyellow and red modules are more close to each other than grey60, brown, blue, turquoise and SCENT (F test; $p<9.8e^{-10}$ between darkolivegreen and SCENT, $p= 2.18e^{-08}$ between violet and SCENT, $p= 5.136e^{-08}$ between greenyellow and SCENT, $p= 1.735e^{-06}$ between grey60 and SCENT, $p= 1.025e^{-05}$ between red and SCENT, $p= 0.0001345$ between brown and SCENT, $p= 0.0001265$ between blue and SCENT) (Supplementary Fig. S3b, Supplementary Table S2). Thus, any stimuli can travel faster in darkolivegreen module than greenyellow module.

Module significance by gene ontology term enrichment

Gene ontology (GO) terms enrichment analyses within blue and turquoise modules of ASCENT were performed with DAVID[9] and Panther[10] to offer a genetic explanation of the constructed gene co-expression network. Both modules were augmented in GO terms associated to explicit Biological Processes (BP). Significance validation of the detected GO term enrichments for both modules was supported by Panther. The blue module having 356 genes with the largest number of enriched terms included enriched GO categories associated with transcriptional regulation, oxidation-reduction process, signal transduction, response to abscisic acid and response to salt stress GO:0006355, GO:0055114, GO:0007165, GO:0009737 and GO:0009651 respectively (Supplementary Fig. S8a). While, the turquoise module, comprised of 632 genes obligated an over-representation of BP terms related to transcriptional regulation, oxidation-reduction process, photosynthesis, defense response, response to cold and response to ABA (e.g., GO:0006355, GO:0055114, GO:0015979, GO:0006952, GO:0009409 and GO:0009737) (Supplementary Fig. S8b, Supplementary Table S3). Additionally, we identified the protein classes encoded by genes in both modules by Panther. It is observed that Nucleic Acid Binding, Oxidoreductase, Hydrolase, Transferase, Ligase and Transporter protein classes were coded abundantly with minimum ten genes in blue module. While, prominent proteins classes encoded by turquoise module genes are Oxidoreductase, Nucleic Acid Binding, Transferase, Hydrolase, Ligase and Transporters (Supplementary Table S3).

Similarly, we pulled out the genes of seven significant modules from SCENT for GO analysis. We formed two module groups; 1st (darkolivegreen, greenyellow, violet) and 2nd (red, grey60, brown and blue). It was observed that genes of the 1st group are mostly involved in oxidation-reduction process (41 genes) followed by protein ubiquitination (25 genes), protein transport and multicellular organism development (17 genes each) and vesicle-mediated transport (11 genes) (Supplementary Fig. S4a, Supplementary Table S3). While, the 2nd group of module genes are involved in protein phosphorylation (101 genes) followed by response to salt stress (71 genes), response to cold (42 genes), protein folding (39 genes), response to water deprivation (38 genes) and abscisic acid-activated signaling pathway (32 genes) (Supplementary Fig. S4b, Supplementary Table S3). Also, panther protein classification signifies hydrolase being the most encoded protein by both module groups followed by transferase, nucleic acid

binding, oxidoreductase, transporter, transcription factor, enzyme modulator, cytoskeletal protein, ligase and lyase. Importantly, it must be noted that DAVID and Panther GO term enrichment classification represents almost similar classification.

Gene regulatory network modeling

A. Transcription factor associated regulatory network

Direct coordination of major switches in gene expression relies on transcription factors (TFs) and their downstream target genes in an intricate fashion. Thus, TFs can play a dominating role in controlling the onset and complete development of the senescence in leaves. TFs bind to particular target genes' regulatory sequences to activate and/or suppress their transcription. Importantly, several senescence associated genes encoding transcription factors have been known; NAC, MYB, AP2/EREBP, WRKY, C2H2 zinc-finger, GRAS, and bZIP being the largest groups of TFs family[11-13]. However, a systems-level study that discovers a comprehensive list of TFs in leaf senescence is still missing. To investigate the TF-targets coordination of 11,453 nuclear genes and 2,226 antisense transcripts across the Arabidopsis leaf lifespan, we examined the significance of TFs in transcriptional regulation.

Among 11,453 genes of Arabidopsis leaf lifespan, 214 TFs are expressed among the all the annotated 2,193 TFs. We utilized our network biology pipeline (Supplementary Fig. S9) to look for the promoter binding site of these TFs. The binding site as well as target gene information is only available for 103 TFs in online databases (AGRIS[14], AthaMap[15], and Plant Cistrome[16]). To determine the TF/target pairs of remaining 111 TFs, we performed a massive TF binding regulatory sequence scanning on 11,453 senescence nuclear genes (Supplementary Fig. S9). First, we generated all possible combinations (forward, reverse, complement, reverse complement along with degeneracy) of nucleotides for binding of respective TF family based on text mining and family associated TF motif search. Second, we retrieved the 1,000 upstream, 5' UTR sequences and AGI transcript of 11,453 genes. Third, 111 TFs regulatory sequence were subjected to blastn[17] against 1,000 upstream, 5' UTR and AGI transcript sequences of 11,453 genes. Finally, 458,302 possible TF/target combinations (Supplementary Table S4) were identified in Arabidopsis leaf lifespan.

Similarly, among 2,226 antisense transcripts, 163 TFs are found among all annotated TFs in Arabidopsis. To determine the TFs binding to the senescence target genes on regulatory sequences we implied the respective TFs to AGRIS[14], AthaMap[15], and Plant Cistrome[16] databases. Out of 163 TFs, 65 TFs and their corresponding target genes were available through aforementioned online resources, while 98 senescence nuclear genes TFs and their targets were not reported on any database. To determine the TF/target pairs of remaining 98 TFs, we performed a massive TF binding regulatory sequence scanning on 2,226 senescence antisense transcripts (Supplementary Fig. S9). First, we generated all possible combinations (forward, reverse, complement, reverse complement along with degeneracy) of nucleotides for binding of respective TF family based on text mining. Second, we retrieved the 1,000

upstream, 5' UTR sequences and AGI transcript of 2,226 antisense transcripts. Third, 98 TFs regulatory sequence were subjected to blastn[17] against 1,000 upstream, 5' UTR sequences and AGI transcript of 2,226 genes. Finally, 204,761 possible TF/target combinations were identified for 163 TFs in 2,226 senescence antisense transcripts (Supplementary Table S4).

We combined co-expression networks (SCENT and ASCENT) and integrated TF/target association data to represent overall TF/target regulation in senescence. Interestingly, we report 220 TFs in Arabidopsis leaf senescence. While, Woo et al.[1] reported only 94 TFs in senescence, we captured more than double TFs of which 190 TFs are identified by our network biology framework (Fig. 2a, Supplementary Table S4). Moreover, our pipeline could be used to simulate any TF-target relationships in leaf senescence since we are providing all the raw data in the supplementary information. Finally, this computational pipeline can be used for other plant-related processes.

Furthermore, we focused our TF/target identification to blue and turquoise modules of ASCENT in order to find the TF affecting network in Arabidopsis leaf senescence. It has been observed that 24 TFs and 48 TFs were present in blue and turquoise modules respectively (Supplementary Table S4). The expression pattern of these TFs in senescence data revealed that only nine TFs are down-regulated at early 'G to M' phase, eight TFs down regulated at transition phase ('G to M' to 'M to S'), while 12 TFs are up-regulated and four TFs are down-regulated at late 'M to S' phase in blue module. Interestingly, 12 TFs are part of hub[75] genes pertaining high connections. Also, TFs of turquoise module followed a similar pattern; 24 TFs are down-regulated at early 'G to M' phase, 18 TFs down regulated and three TFs are up-regulated at transition phase ('G to M' to 'M to S'), while 11 TFs are up-regulated and 18 TFs are down-regulated at late 'M to S' phase in senescence life span. While, two TFs are part of hub[75] genes pertaining high connections. Interestingly, significant greater quantity of differentially expressed TFs at 'M-to-S' phase advocates that Arabidopsis leaves employ the alterations in TFs more dynamically at the onset or late stage of senescence.
Likewise, we focused our TF/target identification to seven modules of SCENT in order to find the TF affecting network in Arabidopsis leaf senescence. It has been observed that 21 TFs are present in red module followed by 12 TFs each in blue and brown modules. While, 11 TFs are present in greenyellow followed by 4 TFs each in darkolivegreen and grey60. Violet modules represent 3 TFs only. Though all TFs are scattered throughout SCENT, interestingly, four TFs are part of hub[500] genes pertaining high connections (ANAC046, ANAC019, ANAC032 and ANAC055) regulating most of the genes (Supplementary Table S4).

B. miRNA associated regulatory network

MicroRNAs (miRNAs;miRs) play significant regulatory role in multiple cellular activities throughout plant developmental progression[18]. It has been reported that miRNAs regulate the genic regulation by the RNA interference process[19,20]. To identify the role

of miRNA regulation in Arabidopsis leaf senescence, we retrieved all 325 high confidence Arabidopsis mature miRs sequences from miRBase miRNA repository[21] and 2,226 antisense transcripts from TAIR online repository[22] in fasta formats. The miR-target identification was performed by psRNATarget online tool[23] with default parameters for small RNA sequences. It is observed that out of 325 miRs, only 60 miRs interact with 115 target genes generating 164 miR-target interactions to establish the functional dynamic miR-based regulatory networks in ASCENT (Supplementary Table S4). Finally, 281 miR-target interactions were predicted on Arabidopsis leaf senescence (SCENT) by aforementioned method (Supplementary Table S4).

Reconstructing senescence responsive dynamic regulatory events

Scalable Models for the Analysis of Regulation from Time Series (SMARTS), a method which incorporates static and time series expression data to reconstruct condition-specific reaction network in an unsupervised manner[24]. First, Time Series Expression Experiments (TSEEs) is associated for datasets representing a collective biological time scale. Next, clustering is utilized to acquire a preliminary assembly for the TSEEs using associated time series. Finally, several iterations are executed to create regulatory models for sets of TSEEs and iteratively conveying TSEEs to models until convergence is achieved. Additionally, the regulatory model identifies specific stimulated pathways and genes, which uses statistical analysis to recognize TFs that vary in activity among models. Also, SMARTS can integrate miRs Dynamic Regulatory Events Miner (mirDREM)[25], a unique miR time series expression measurement and miR-target genes for dynamic miR-regulated interaction networks. We implemented SMARTS[24] on 11,453 nuclear genes expression pattern with $\log_2$ normalization for dynamic regulatory event mining with all possible 458,302 TF/target and 281 miR-target interactions (SCENT, Fig. 1d, Supplementary Table S5).The miR expression data was retrieved from[1]. The dynamic activated pathways regulated by TFs and miR was generated by TAIR GO ontology function. Also, we executed SMARTS[24] on 204,761 possible TF/target combinations and 164 miR-target interactions for 2,226 AGI antisense transcripts (ASCENT) and complete leaf senescence expression profile with $\log_2$ normalization (Supplementary Fig. S10a, Supplementary Table S5). In ASCENT, this analysis modeled 22 different paths that are governed by 11 different groups of TFs and several miRs (Supplementary Fig. S10A, Supplementary Table S5, significance threshold = 0.040 for TFs and 0.251 for miRs).

Gene regulatory network simulation

Standardized Qualitative Dynamical systems (SQUAD)[26] executes steady state studies and dynamic simulations of genes and their interacting TFs by highlighting system equilibrium and their fluctuations upon regulatory stimuli (TF). The activity significance of each node was fixed to zero (initial steady state) representing the preliminary point for continual dynamic simulations to achieve the equilibrium state for network. The reciprocated impression of a stimuli (TF) is studied by modulating the initial activation to 1. Additionally, the partial initiation for input stimuli was adjusted between 0 and 1. SQUAD alter the outcome of these regulation into a time-fixed

sigmoid activity curve for all connecting nodes of the network which can be visualized by trajectory plots. We implemented SQUAD simulation on integrated TF-target directional regulatory network ASCENT consisting of 237 nodes with 282 connections and SCENT containing 2,637 nodes and 5,020 connections; a total of 5,295 TF/target co-regulation (Fig. 2a). Here we display the activation states of two representative TFs *i.e.* ANAC046 and AT1G71520. The activation state of TF (AT1G71520) was changed from 0 to 1 to simulate the resulting trajectory plots of 22 putative target genes according to logical connectivity including partial activation of nodes (Supplementary Fig. S10b, Supplementary Table S6). ANAC046 co-regulates with 137 genes in SCENT among which, 49 nodes exhibit hub[800] property in SCENT, whereas AT1G71520 co-regulates with 22 genes in ASCENT of which 18 nodes exhibit hub[75] property. 18 of these 49 targets of ANAC046 (Supplementary Fig. S10c, Supplementary Table S6), and 10 of 18 targets of AT1G71520 have been reported in autophagy or senescence-associated processes. The activation of ANAC046 and AT1G71520 over time was used as an index to measure the behavior of its putative downstream target genes (Supplementary Fig. S10b and c; Supplementary Table S6). This analysis displayed varied levels of activation for these 137 and 22 genes for ANAC046 and AT1G71520 respectively. Schematic representation of target genes that are regulated by only ANAC046 and AT1G71520 or in combination with ANAC046, AT1G71520 and other TFs estimated through Regulatory Network Simulation by SQUAD (Fig. 2B). Similar to these two TFs, SQUAD can be applied to all the 210 TFs and 163 TFs for SCENT and ASCENT, respectively.

Plant material and growth conditions

Arabidopsis thaliana seeds (Col-0) were sown on soil and stratified for 3 days at 4°C. The seeds were then transferred to growth conditions (12h light / 12h dark at 22°C). Dark-induced senescence was conducted as previously described in[27]. Briefly, the fifth or sixth leaves of four-week old plants were detached in placed on two filter papers soaked in 10mL of distilled water in a petri dish. The petri dishes were incubated in the dark at 22°C.

Protein extraction and Western blotting

For each reported day, leaves were frozen in liquid nitrogen and ground using a mortar and pestle. Protein was extracted from leaf tissue in a buffer containing 50mM HEPES, 10mM EDTA, 50mM NaCl, 0.5% (v/v) Triton X-100, 5mM DTT, 5% glycerol (v/v), protease inhibitor (1x; P8340; Sigma), 1mM PMSF. The subsequent slurry was centrifuged at 13,000 rpm for 20 minutes at 4°C. 75µL of the supernatant was mixed with 25µL of 4x sample buffer (NP0008; Thermo Fisher Scientific) and boiled at 100°C for 5 minutes. After 2 minutes of ice the samples were centrifuged at 13,000 rpm for 2 minutes. The resulting supernatant was loaded onto a 10% SDS-PAGE gel. SAG12 was visualized after transfer using a SAG12 specific antibody (AS14 2771; Agrisera) (Fig. 2c).

Fv/Fm quantification

Fv/Fm was used as an indicator of senescence by accessing the efficiency of photosystem II (PSII). Using genetics approach, it was shown that ANAC046 participates in leaf senescence[28]. Transgenic plants overexpressing ANAC046 (ANAC046-OX) and ANAC046 containing a SRDX domain (ANAC046-SRDX) were previously described[28]. Eight detached leaves from Col-0, ANAC046-OX, and ANAC046-SRDX were dark adapted for 15 minutes and measurements were acquired using a chlorophyll fluorometer (OS-30p+ Opti-Sciences). Measurements were taken immediately after detachment of eight leaves from four different plants of above described genotypes on days 2, 3, and 6. A two-tailed Students t-test was applied to determine statistical significance. The ANAC046-SRDX line had a p-value of 0.03 on day 6 when compared to day 6 Col-0. We determine the efficiency of the PSII at each time point using a chlorophyll fluorometer to measure Fv/Fm (Fig. 2c). At day 6, we show the ANAC046-SRDX plants is significantly higher than Col-0 (p-value 0.03), while ANAC046-OX showed reduced efficiency of PSII, although not statistically significant. These data together indicate that ANAC046 is a positive regulator of leaf senescence.

RNA extraction and quantitative real time PCR (qRT-PCR)

Total RNA from leave samples was extracted with RiboZol™ RNA Extraction Reagent (AMRESCO), followed with DNase I treatment to remove any Genomic DNA contamination. Complementary DNA (cDNA) was synthesized with the SuperScript III first-strand RT-PCR kit (Invitrogen). qRT-PCR (Supplementary Fig. S11) was performed with gene specific primers (Supplementary Table S7) and GoTaq qPCR Master Mix (Promega) in a RealPlex S MasterCycler (Eppendorf).

Mathematical model of qRT-PCR data by $C_{\gamma 0}$ method

$C_{\gamma 0}$ is the first method in Real-time PCR analysis that does not require the assumption of equal efficiency between unknowns and the standard curve. The point of intersection between the x-axis and tangent through the inflection point of the PCR data plot is $C_{\gamma 0}$.

The $C_{\gamma 0}$ was calculated by fitting of the mathematical function, Richards's equation, to fit the raw PCR florescence data[29,30].

Richards mathematical function:

$$Fx = f(x; Fmax, b, c, d) = Fbrf + \frac{Fmax}{\left[1+e^{-\frac{1}{b}(x-c)}\right]^{d}} \qquad (1)$$

Where,

x is the PCR cycle number, F_x is fluorescence at cycle *x*, F_{max} is the maximum fluorescence, *d* is the Richards coefficient, and F_{brf} is the background reaction fluorescence.

The inflection point (F_{lex}) was calculated as follows:

$$Flex = \left[c + b\ln d\ ; Fmax\left(\frac{1}{d+1}\right)^{d} + Fbrf\right] \qquad (2)$$

And the $C_{\gamma 0}$ was calculated as follows:

$$C\gamma 0 = c + b\ln d - b\left(\frac{d+1}{d}\right)\left[1 - \frac{Fbrf}{Fmax}\left(\frac{d+1}{d}\right)^{d}\right] \qquad (3)$$

$C_{\gamma 0}$ value is inversely proportional to amount of cDNA in the reaction and reflects the reaction kinetics since it is derived through slope of the inflection point of the raw fluorescence data. For example, $C_{\gamma 0}$ for SAG12 in ANAC046-OX (overexpressor) is lower than both Col-0 (wild type) and ANAC046-SRDX (knockout) at day 6, which illustrates that ANAC046-OX is kinetically more active than the Col-0 or ANAC046-SRDX. Similar $C_{\gamma 0}$ kinetics was observed for AT5G40690 transcript accumulation in the abovementioned genotypes (Fig. 2e and f).

Raw datasets was fitted to the mathematical model (Equation 1), and $C_{\gamma 0}$ (Equation 3) was calculated if R^2 was greater than 0.997. All calculations were done using in house python scripts.

Supplementary Discussion

Senescence is a multilayered and extremely organized aging process in plants' life cycle[1]. Plants employ a wide range of regulators that reprogram regulatory components in a highly synchronized manner to properly execute this complex developmental stage of plant life. On the contrary, the static regulatory network models demonstrate the direct transcriptional regulation without any information of time/stage specific regulation or molecular events/rewiring followed through regulators[24]. Therefore, revealing the dynamic transcriptional regulation during the onset of senescence is essential for a compressive understanding of temporal relationships among TFs and their targets. Towards this, we developed a computationally intensive pipeline to predict the regulatory networks that integrates publically available tools to study the regulation dynamics as well as the behavior of master regulators and their downstream target genes by computational simulations. With limited availability of TFs binding site information in the public domain, our sequence and co-expression based TF binding site prediction method can enrich the TF-target associations. By employing this method, we enriched the TFs-target prediction by over two folds in this study. Furthermore, the implementation of predicted TF-target association, miRs-terget interactions and transcriptome expression can reveal the stage specific dynamics of TFs and miRs regulation to predict the changes in functional association of genes. Finally, the simulated response of downstream targets and master regulators predictions based on network topology measures and ODE models can be useful for laboratory experimental validations, which will eventually reduce the time and cost of experimentation. Our

computational pipeline can be an ideal model to the scientific community for predicting the TFs binding motifs and application of transcriptional regulatory networks to determine the dynamics of regulation to explore diverse plant biology challenges like abiotic and biotic stresses in crop plants.

Supplementary Figures

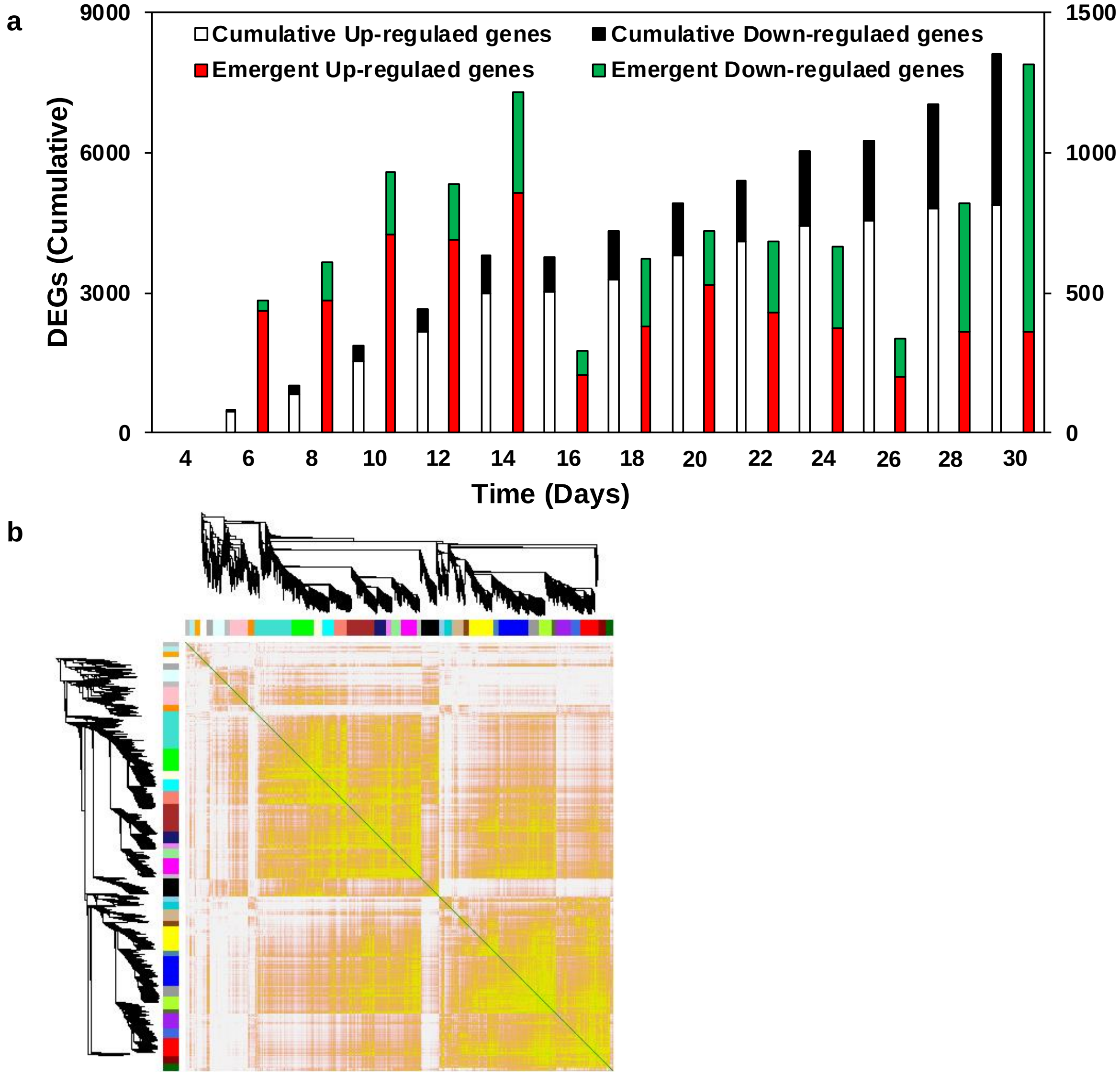

Supplementary Fig. S1

Gene Expression analyses for nuclear encoded genes in senescence.

(**a**) Gene Expression analyses of nuclear genes reveals important Emergent DEGs. Primary and secondary axes represent cumulative and emergent DEGs respectively. Blue and green colored columns represent cumulative up-regulated DEGs while red and brown colored columns represent emergent up-regulated and down-regulated DEGs on each day during Arabidopsis leaf senescence.

(**b**) Weighted co-expressed network construction of Arabidopsis leaf senescence DEGs. Topological overlap mapping metric (TOM) heatmap based hierarchical clustering for co-expressed genes in each module within the SCENT (Senescence CoExpression NeTwork). The intensity of yellow followed by pink colors denotes the strength of clustering.

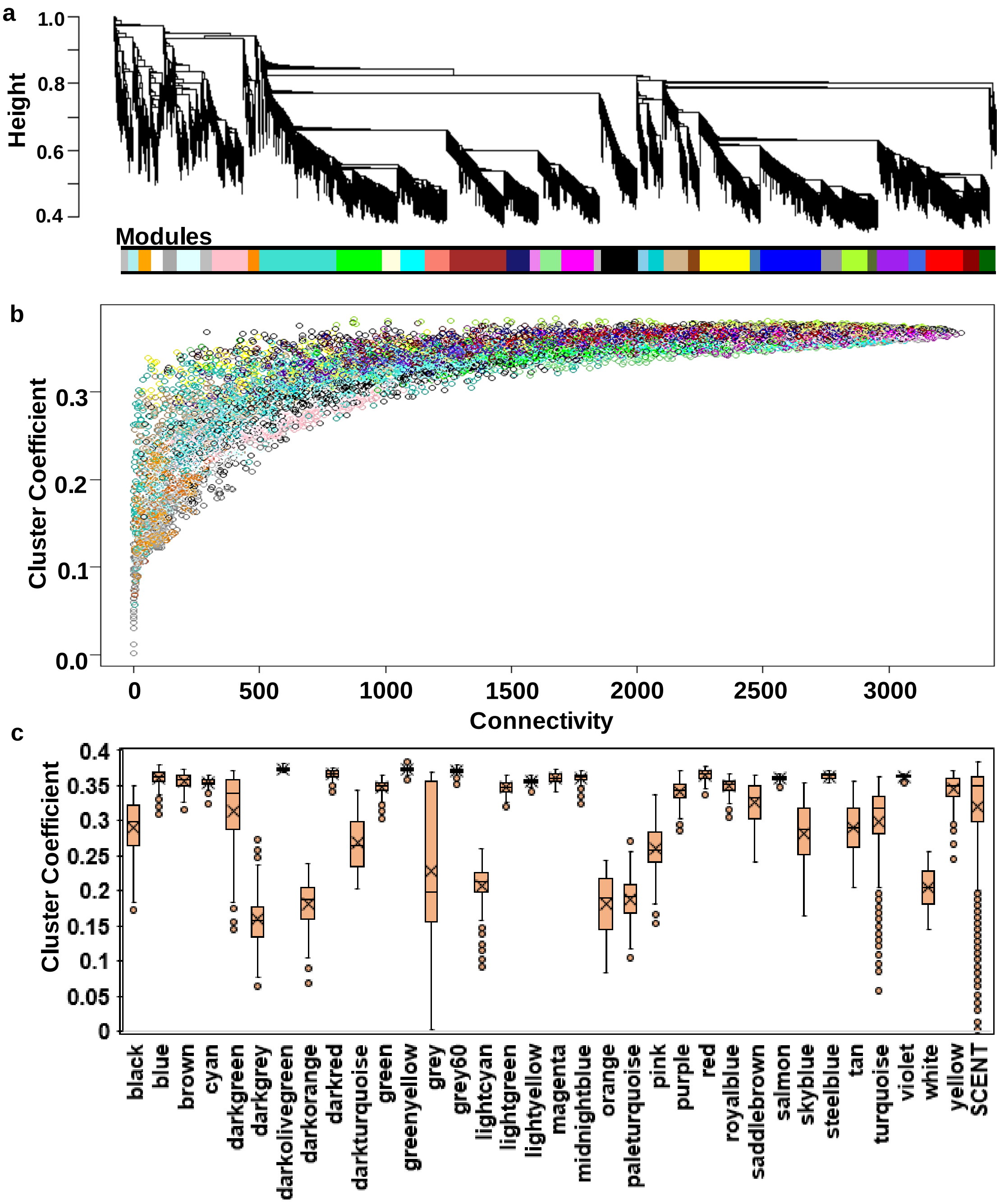

Supplementary Fig. S2

Module identification using Weighted Gene Co-expression Network Analysis.

(**a**) 34 modules generated by WGCNA in SCENT (Senescence CoExpression NeTwork). We ignored “grey” module as it has the lowest clustering coefficient.

(**b**) The cluster coefficient VS connectivity plot of co-expressed gene with colored modules in SCENT.

(**c**) Seven modules out of 33 in SCENT exhibit heightened connectivity and average cluster coefficient compared to other modules or SCENT are darkolivegreen, greenyellow, violet, red, grey60, brown, blue and turquoise.

a

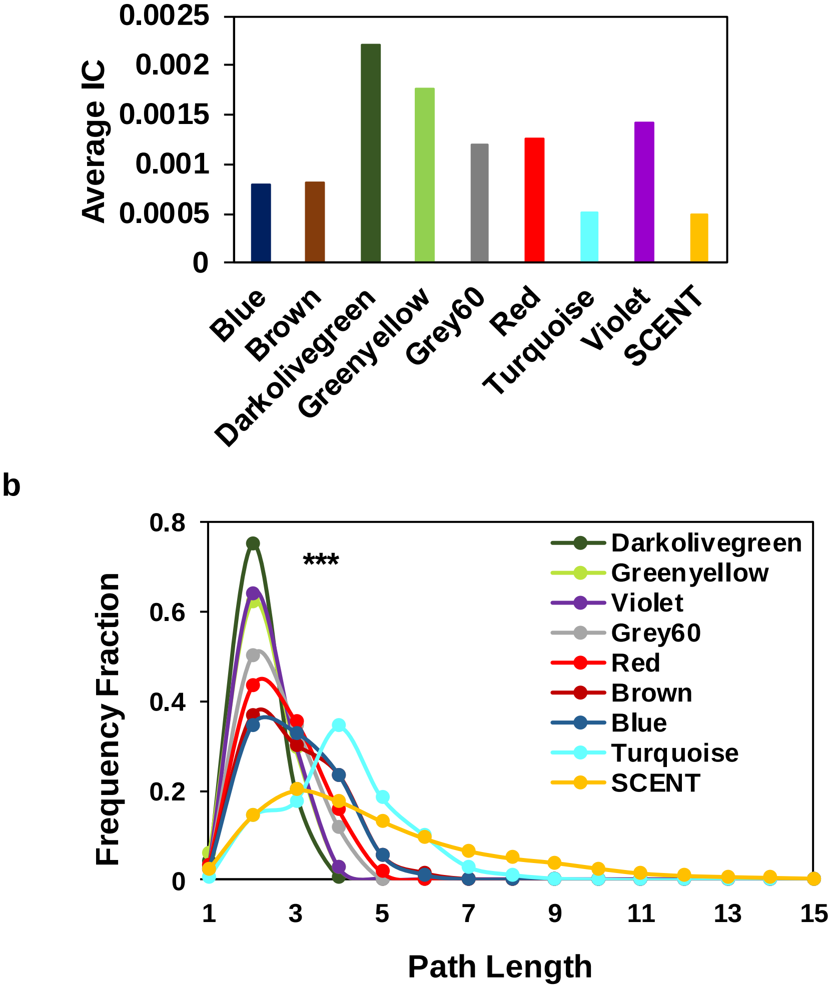

Supplementary Fig. S3

Network topology analyses in SCENT (Senescence CoExpression NeTwork).

(**a**) Average Information Centrality (IC) measure of darkolivegreen, greenyellow, violet, red, grey60, brown, blue and turquoise modules genes and SCENT. Darkolivegreen, greenyellow, violet, red, and grey60 modules have a higher average IC than SCENT.

(**b**) Shortest path distribution reveals that genes in the darkolivegreen, violet, greenyellow and red modules are more close to each other than grey60, brown, blue, turquoise and SCENT (F test; $p<9.8e^{-10}$ between darkolivegreen and SCENT, p= $2.18e^{-08}$ between violet and SCENT, p= $5.136e^{-08}$ between greenyellow and SCENT, p= $1.735e^{-06}$ between grey60 and SCENT, p= $1.025e^{-05}$ between red and SCENT, p= 0.0001345 between brown and SCENT, p= 0.0001265 between blue and SCENT).

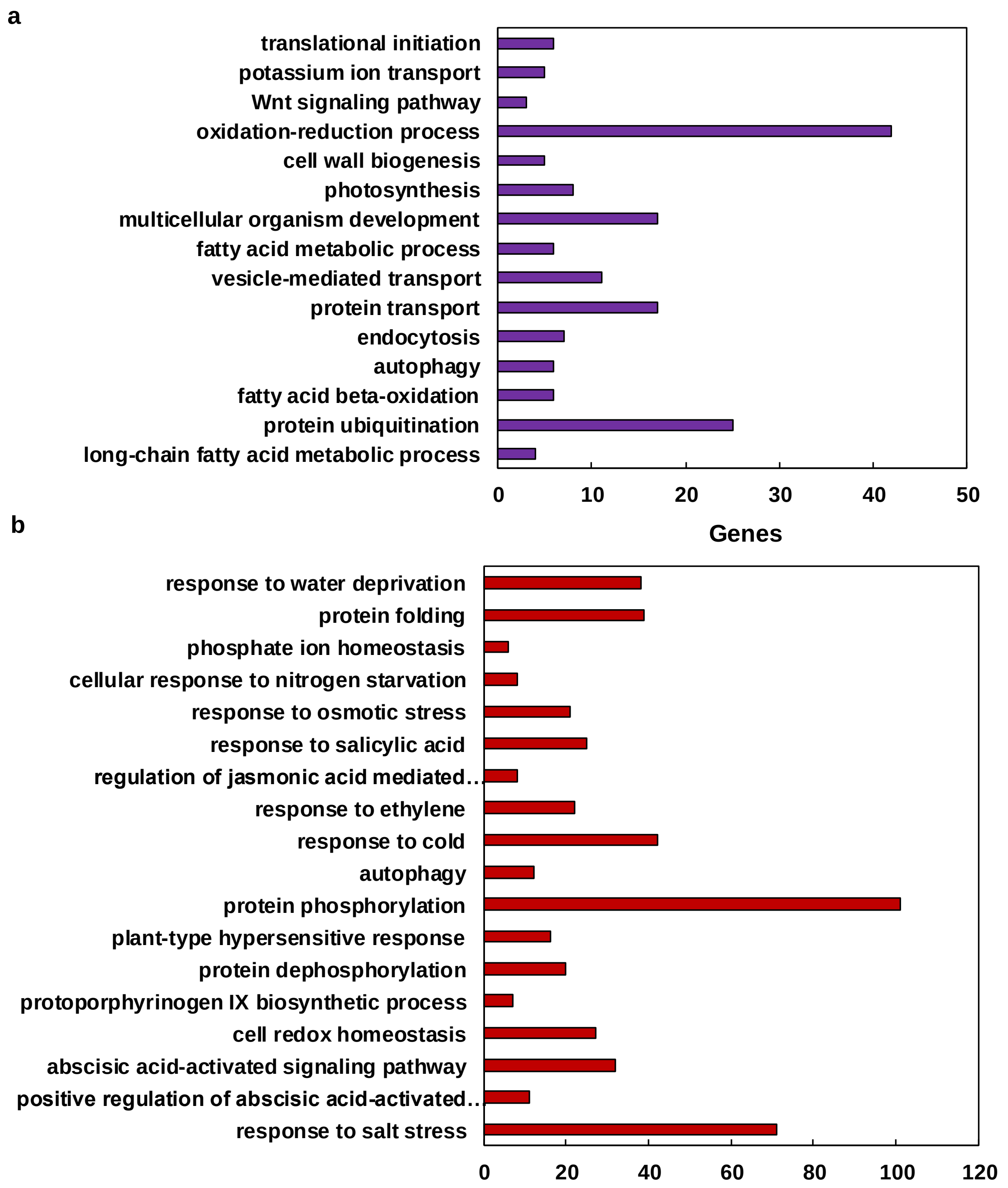

Supplementary Fig. S4

Module significance by gene ontology term enrichment in SCENT (Senescence CoExpression NeTwork).

(**a**) Darkolivegreen, greenyellow, violet module genes are mostly involved in oxidation-reduction process (41 genes) followed by protein ubiquitination (25 genes), protein transport and multicellular organism development (17 genes each) and vesicle-mediated transport (11 genes).

(**b**) Red, grey60, brown and blue module genes are involved in protein phosphorylation (101 genes) followed by response to salt stress (71 genes), response to cold (42 genes), protein folding (39 genes), response to water deprivation (38 genes) and abscisic acid-activated signaling pathway (32 genes).

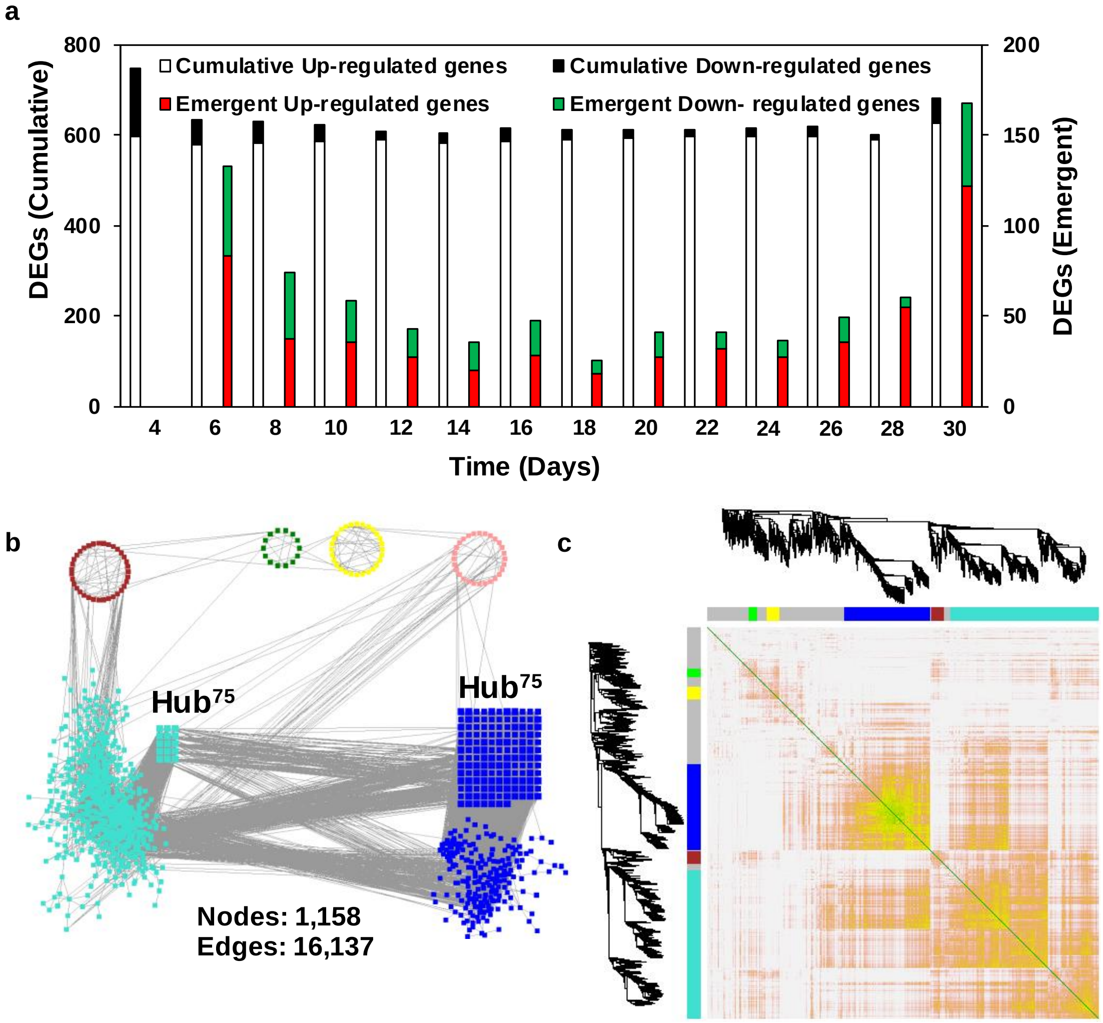

Supplementary Fig. S5

Gene Expression analyses for antisense transcripts in senescence.

(**a**) Gene Expression analyses reveals important Emergent antisense DEGs. Primary and secondary axes represent cumulative and emergent DEGs respectively. Blue and green colored columns represent cumulative up-regulated DEGs while red and brown colored columns represent emergent up-regulated and down-regulated antisense transcripts on each day during Arabidopsis leaf senescence.

(**b**) Weighted co-expressed network construction of antisense Arabidopsis leaf senescence DEGs. A weighted co-expression network with weight ≥0.75 constructed by WGCNA was visualized in cytoscape containing 1,158 co-expressed genes (nodes) with 16,137 interactions (edges) and five modules representing the entire ASCENT (Antisense Senescence CoExpression NeTwork).

(**c**) Weighted Gene Co-expression network was constructed and five significant modules were identified with minimum module size >50 represented in topological overlap mapping metric (TOM) heatmap based hierarchical clustering for co-expressed genes in each module within the ASCENT. The intensity of yellow and pink colors denotes the strength of clustering.

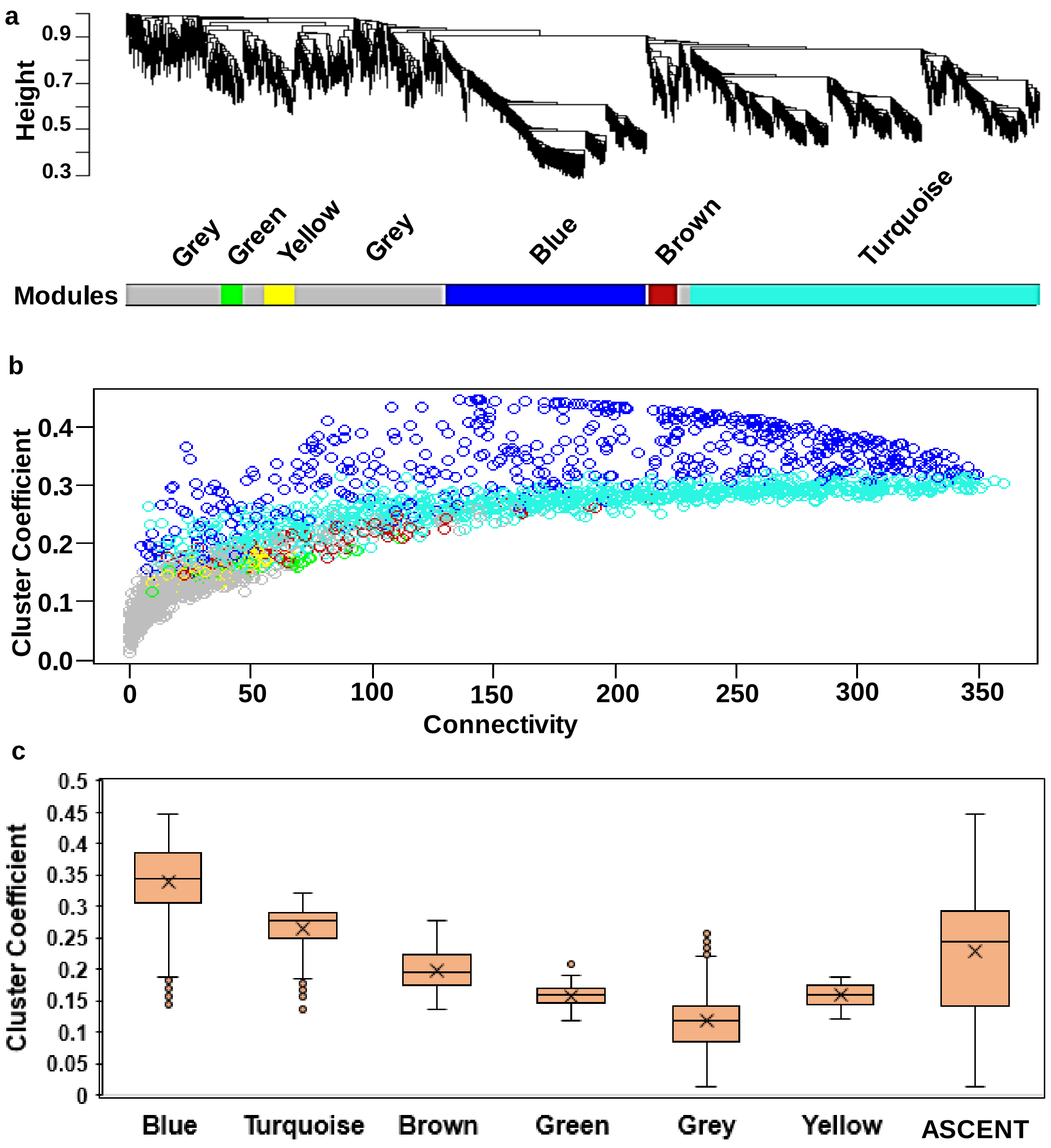

Supplementary Fig. S6

Module identification using Weighted Gene Co-expression Network Analysis.

(**a**) Seven modules generated by WGCNA in ASCENT (Antisense Senescence CoExpression NeTwork). We ignored "grey" module as it has the lowest clustering coefficient.

(**b**) The cluster coefficient VS connectivity plot of co-expressed gene with colored modules in ASCENT.

(**c**) Average cluster coefficient of each modules and ASCENT. Blue and turquoise modules have a significantly higher average cluster coefficient than ASCENT. While, 694 genes were not assigned to any of the five modules with the lowest cluster coefficient thus supplemented to a 'grey' (or insignificant) module.

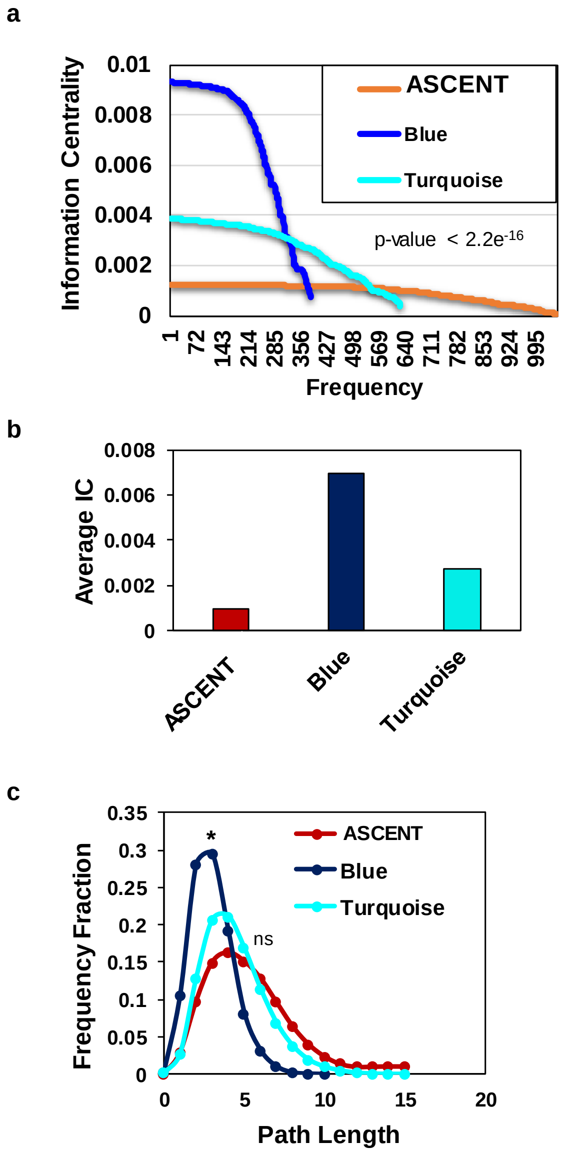

Supplementary Fig. S7

Network topology analyses in ASCENT (Antisense Senescence CoExpression NeTwork).

(**a**) Information centrality (IC) measure of blue and turquoise modules are significantly high in the largest component of co-expression network. The Students t-test (p-value < $2.2e^{-16}$) was applied to check the significance ICs of blue and turquoise module against ASCENT.

(**b**) Average Information Centrality (IC) measure of blue, turquoise modules genes and whole senescence genes. Blue module has highest average IC followed by turquoise than ASCENT.

(**c**) Shortest Path Length analysis of blue and turquoise modules reveals that blue module genes have more close connections than turquoise and ASCENT (F.test, P= 0.020970693 between Blue and ASCENT, F.test, P= 0.295933097 between turquoise and ASCENT).

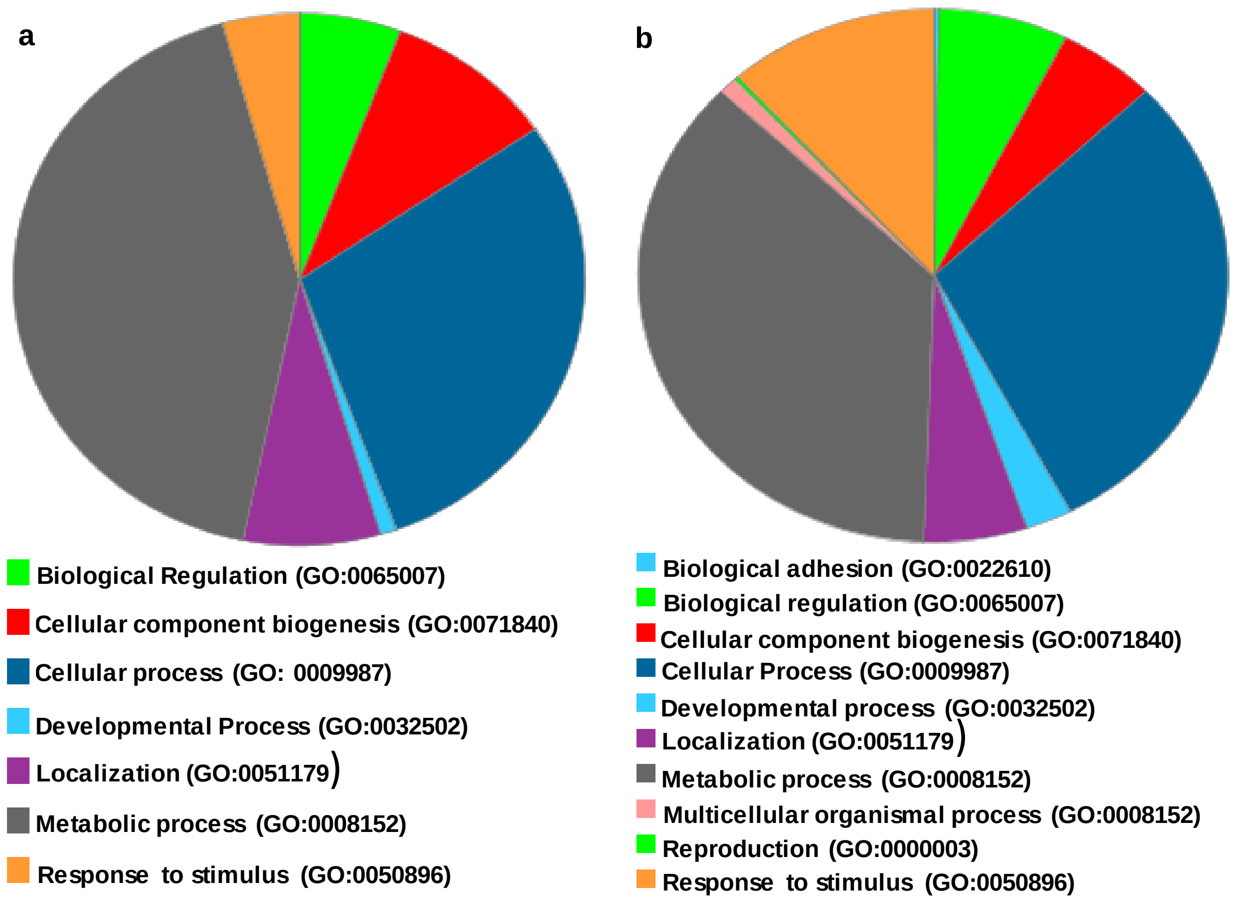

Supplementary Fig. S8

Module significance by gene ontology term enrichment in Blue and Turquoise modules in ASCENT (Antisense Senescence CoExpression NeTwork).

(**a**) Blue module genes biological processes illustrate more genes involved in metabolic, cellular, cellular component biogenesis and localization process.

(**b**) Turquoise module genes biological processes illustrate more genes involved in metabolic, cellular, response to stimulus and reproduction processes.

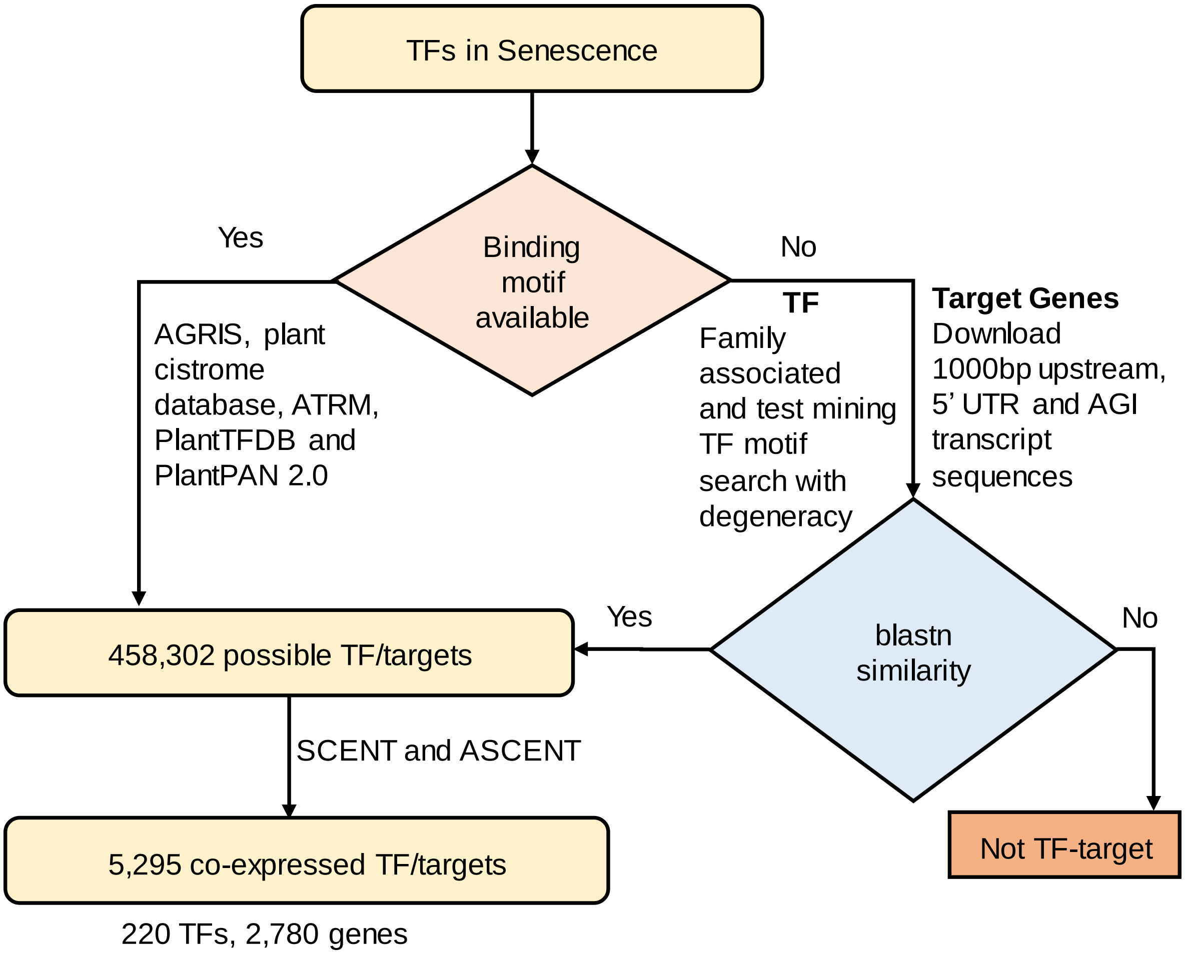

Supplementary Fig. S9

Identification of Transcription factors (TFs) in leaf senescence.

To determine the TF/target pairs of TFs whose promoter binding sites are unknown, we performed a massive TF binding regulatory sequence scanning on senescence nuclear genes. First, we generated all possible combinations (forward, reverse, complement, reverse complement along with degeneracy) of nucleotides for binding of respective TF family based on text mining and family associated TF motif search. Second, we retrieved the 1000bp upstream and 5' UTR and AGI sequences of senescence nuclear genes. Third, TFs regulatory sequence were subjected to standalone blastn against 1,000 upstream 5' UTR and AGI sequences of senescence nuclear genes. 458,302 possible TF/target combinations were identified, finally we integrated coexpression networks to get the high confidence TF/targets for 220 TFs senescence.

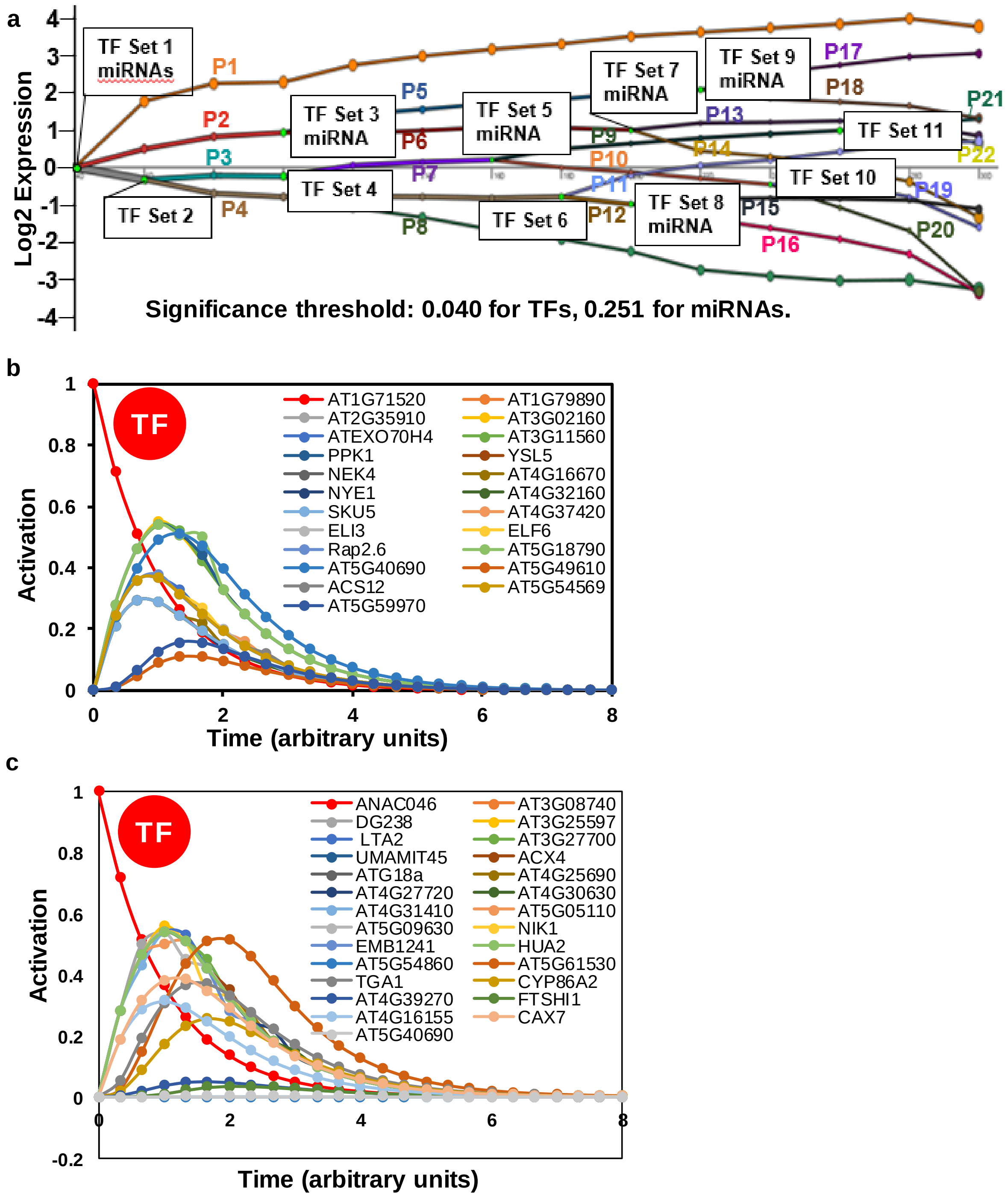

Supplementary Fig. S10

Gene regulatory models and simulations in leaf senescence.

(**a**) Reconstructed Responsive Dynamic Regulatory Events for antisense transcripts (ASCENT; Antisense Senescence CoExpression NeTwork) modeled by SMARTS (Scalable Models for the Analysis of Regulation from Time Series). Modeled TF/target gene interactions, miRNA-target gene interaction, senescence gene expression and miRNAs expression values were utilized for event mining. The colored lines represent clustered genes based on their expression pattern and attach the median regulated

gene clusters. The green nodes denote a set of genes, mutually regulated till that interval split. The responsive group of TFs and miRNAs are listed in Supplementary Table S5. The dynamic activated pathways regulated by TFs and miRNA was generated by TAIR GO ontology function, represented by a particular color with path numbers. This analysis modeled 22 different paths that are governed by 11 different groups of TFs and several miRNAs (Supplementary Fig. S10A, Supplementary Table S5, significance threshold = 0.040 for TFs and 0.251 for miRNAs).
(**b**) Senescence Regulatory Network Simulation by SQUAD (Standardized Qualitative Dynamical systems). The simulated steady state regulatory network trajectory plots were modeled according to logical connectivity including partial activation of nodes. The change in activation state of TF (AT1G71520) from 0 to 1 stimulated the resulting trajectory plots of 22 putative target genes according to the connectivity of partial activated nodes.
(**c**) Senescence Regulatory Network Simulation by SQUAD. The simulated steady state regulatory network trajectory plots were modeled according to logical connectivity including partial activation of nodes. The change in activation state of TF (ANAC046) from 0 to 1 stimulated the resulting trajectory plots of 27 putative target genes out of 127 (for clear visualization) according to the connectivity of partial activated nodes.

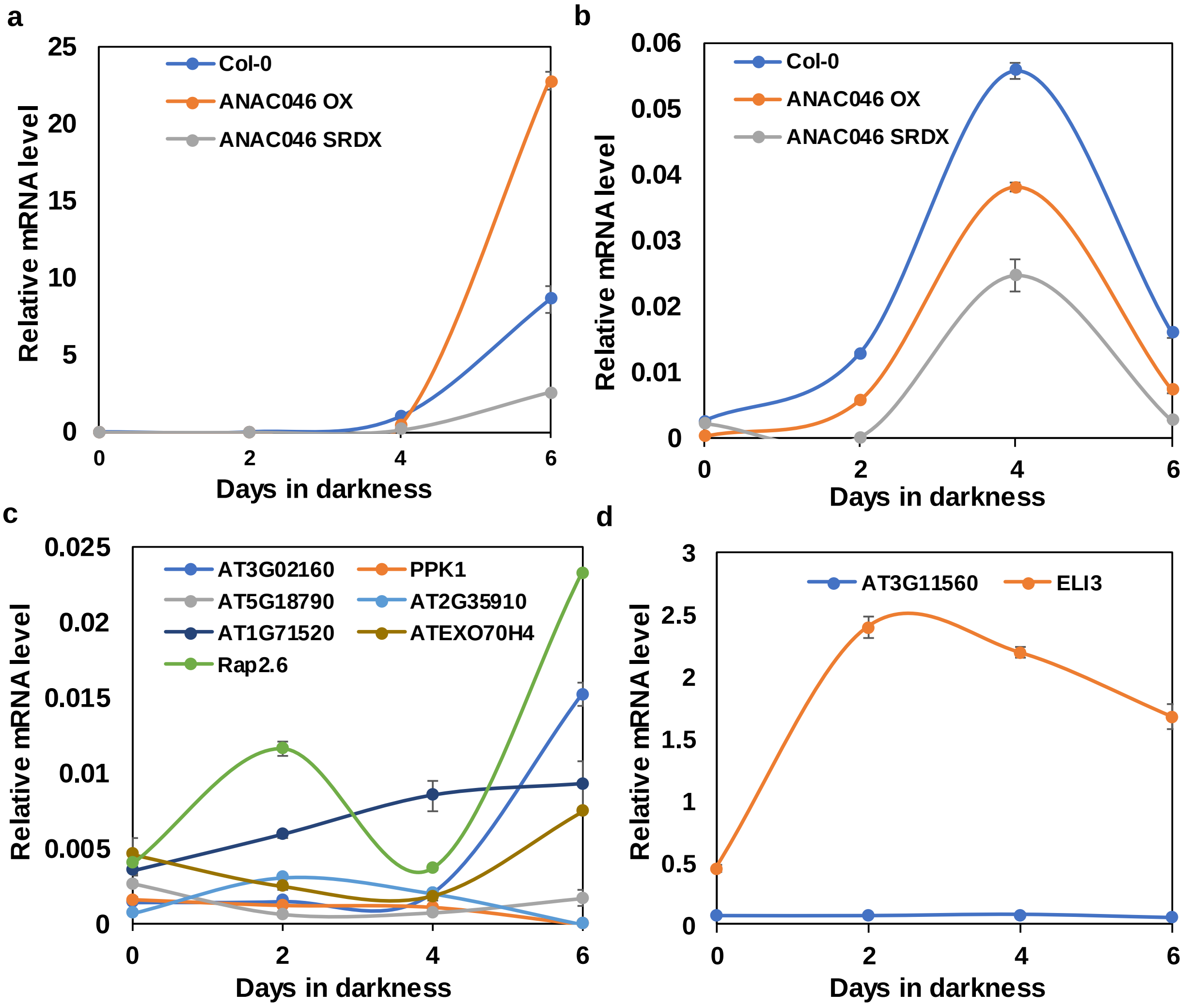

Supplementary Fig. S11

Differential expression of genes in Col-0, ANAC046-OX, and ANAC046-SRDX lines upon dark treatment.

(**a**) Upon dark treatment, SAG12 transcripts accumulate differentially in Col-0, ANAC046-OX and ANAC046-SRDX lines. Pronounced transcript levels can be observed at day 6 in ANAC046-OX compared to Col-0 and ANAC046-SRDX.

(**b**) The transcript levels of AT5G40690 are observed in Col-0, ANAC046-OX and ANAC046-SRDX lines upon dark treatment at the indicated time points.

(**c and d**) Differential increase of transcript for the indicated transcription factors at different time points were shown. No significant change in PPK1 and AT3G11560 transcript levels was observed.

Supplementary Tables

Supplementary Table S1: Gene Expression pattern analyses

Supplementary Table S2: Co-expressed Networks construction and analyses

Supplementary Table S3: Module significance by GO enrichment analyses

Supplementary Table S4: TF/target & miRNA-target modeling

Supplementary Table S5: Reconstruction of Senescence Responsive Dynamic Regulatory Events

Supplementary Table S6: Senescence co-expressed Gene Regulatory Network Simulation

Supplementary Table S7: Primers used for qRT-PCR

Supplementary References

1 Woo, H. R. *et al.* Programming of Plant Leaf Senescence with Temporal and Inter-Organellar Coordination of Transcriptome in Arabidopsis. *Plant physiology* **171**, 452-467, doi:10.1104/pp.15.01929 (2016).

2 Barrett, T. & Edgar, R. Mining microarray data at NCBI's Gene Expression Omnibus (GEO)*. *Methods in molecular biology* **338**, 175-190, doi:10.1385/1-59745-097-9:175 (2006).

3 Tully, J. P. *et al.* Expression-based network biology identifies immune-related functional modules involved in plant defense. *BMC genomics* **15**, 421, doi:10.1186/1471-2164-15-421 (2014).

4 Langfelder, P. & Horvath, S. WGCNA: an R package for weighted correlation network analysis. *BMC bioinformatics* **9**, 559, doi:10.1186/1471-2105-9-559 (2008).

5 Zhang, B. & Horvath, S. A general framework for weighted gene co-expression network analysis. *Statistical applications in genetics and molecular biology* **4**, Article17, doi:10.2202/1544-6115.1128 (2005).

6 Saito, R. *et al.* A travel guide to Cytoscape plugins. *Nat Methods* **9**, 1069-1076, doi:10.1038/nmeth.2212 (2012).

7 Hagberg, A., Swart, P. & S Chult, D. Exploring network structure, dynamics, and function using NetworkX. (Los Alamos National Laboratory (LANL), 2008).

8 Brandes, U. & Fleischer, D. in *STACS 2005: 22nd Annual Symposium on Theoretical Aspects of Computer Science, Stuttgart, Germany, February 24-26, 2005. Proceedings* (eds Volker Diekert & Bruno Durand) 533-544 (Springer Berlin Heidelberg, 2005).

9 Huang da, W., Sherman, B. T. & Lempicki, R. A. Systematic and integrative analysis of large gene lists using DAVID bioinformatics resources. *Nat Protoc* **4**, 44-57, doi:10.1038/nprot.2008.211 (2009).

10 Mi, H. *et al.* PANTHER version 11: expanded annotation data from Gene Ontology and Reactome pathways, and data analysis tool enhancements. *Nucleic Acids Res* **45**, D183-D189, doi:10.1093/nar/gkw1138 (2017).

11 Buchanan-Wollaston, V. *et al.* Comparative transcriptome analysis reveals significant differences in gene expression and signalling pathways between developmental and dark/starvation-

induced senescence in Arabidopsis. *Plant J* **42**, 567-585, doi:10.1111/j.1365-313X.2005.02399.x (2005).

12 Gregersen, P. L. & Holm, P. B. Transcriptome analysis of senescence in the flag leaf of wheat (Triticum aestivum L.). *Plant Biotechnol J* **5**, 192-206, doi:10.1111/j.1467-7652.2006.00232.x (2007).

13 Balazadeh, S., Riano-Pachon, D. M. & Mueller-Roeber, B. Transcription factors regulating leaf senescence in Arabidopsis thaliana. *Plant Biol (Stuttg)* **10 Suppl 1**, 63-75, doi:10.1111/j.1438-8677.2008.00088.x (2008).

14 Yilmaz, A. *et al.* AGRIS: the Arabidopsis Gene Regulatory Information Server, an update. *Nucleic Acids Res* **39**, D1118-1122, doi:10.1093/nar/gkq1120 (2011).

15 Bulow, L., Brill, Y. & Hehl, R. AthaMap-assisted transcription factor target gene identification in Arabidopsis thaliana. *Database (Oxford)* **2010**, baq034, doi:10.1093/database/baq034 (2010).

16 O'Malley, R. C. *et al.* Cistrome and Epicistrome Features Shape the Regulatory DNA Landscape. *Cell* **165**, 1280-1292, doi:10.1016/j.cell.2016.04.038 (2016).

17 Altschul, S. F., Gish, W., Miller, W., Myers, E. W. & Lipman, D. J. Basic local alignment search tool. *J Mol Biol* **215**, 403-410, doi:10.1016/S0022-2836(05)80360-2 (1990).

18 Vaucheret, H. Post-transcriptional small RNA pathways in plants: mechanisms and regulations. *Genes Dev* **20**, 759-771, doi:10.1101/gad.1410506 (2006).

19 Elbashir, S. M. *et al.* Duplexes of 21-nucleotide RNAs mediate RNA interference in cultured mammalian cells. *Nature* **411**, 494-498, doi:10.1038/35078107 (2001).

20 Wang, X. B. *et al.* The 21-nucleotide, but not 22-nucleotide, viral secondary small interfering RNAs direct potent antiviral defense by two cooperative argonautes in Arabidopsis thaliana. *Plant Cell* **23**, 1625-1638, doi:10.1105/tpc.110.082305 (2011).

21 Kozomara, A. & Griffiths-Jones, S. miRBase: annotating high confidence microRNAs using deep sequencing data. *Nucleic Acids Res* **42**, D68-73, doi:10.1093/nar/gkt1181 (2014).

22 Lamesch, P. *et al.* The Arabidopsis Information Resource (TAIR): improved gene annotation and new tools. *Nucleic Acids Res* **40**, D1202-1210, doi:10.1093/nar/gkr1090 (2012).

23 Dai, X. & Zhao, P. X. psRNATarget: a plant small RNA target analysis server. *Nucleic Acids Res* **39**, W155-159, doi:10.1093/nar/gkr319 (2011).

24 Wise, A. & Bar-Joseph, Z. SMARTS: reconstructing disease response networks from multiple individuals using time series gene expression data. *Bioinformatics* **31**, 1250-1257, doi:10.1093/bioinformatics/btu800 (2015).

25 Schulz, M. H. *et al.* Reconstructing dynamic microRNA-regulated interaction networks. *Proc Natl Acad Sci U S A* **110**, 15686-15691, doi:10.1073/pnas.1303236110 (2013).

26 Di Cara, A., Garg, A., De Micheli, G., Xenarios, I. & Mendoza, L. Dynamic simulation of regulatory networks using SQUAD. *BMC bioinformatics* **8**, 462, doi:10.1186/1471-2105-8-462 (2007).

27 Yu, J. *et al.* JAZ7 negatively regulates dark-induced leaf senescence in Arabidopsis. *J Exp Bot* **67**, 751-762, doi:10.1093/jxb/erv487 (2016).

28 Oda-Yamamizo, C. *et al.* The NAC transcription factor ANAC046 is a positive regulator of chlorophyll degradation and senescence in Arabidopsis leaves. *Scientific reports* **6**, 23609, doi:10.1038/srep23609 (2016).

29 Guescini, M., Sisti, D., Rocchi, M. B., Stocchi, L. & Stocchi, V. A new real-time PCR method to overcome significant quantitative inaccuracy due to slight amplification inhibition. *BMC bioinformatics* **9**, 326, doi:10.1186/1471-2105-9-326 (2008).

30 Richards, F. J. A Flexible Growth Function for Empirical Use. *Journal of Experimental Botany* **10**, 290-301, doi:10.1093/jxb/10.2.290 (1959).
